# Effect of baduanjin on the fall and balance function in middle-aged and elderly people: A protocol for systematic review and meta-analysis

**DOI:** 10.1097/MD.0000000000027250

**Published:** 2021-09-17

**Authors:** Yao Xiao, Qin Luo, Yunyang Yu, Biwei Cao, Miao Wu, Yujun Luo, Yan Zhao, Jing Zhou

**Affiliations:** a Department of Tuina and Rehabilitation Medicine, Hubei Provincial Hospital of Traditional Chinese Medicine, Wuhan, Hubei, China; b Hubei Academy of Traditional Chinese Medicine, Wuhan, Hubei, China; c Hubei University of Chinese Medicine, Wuhan, Hubei, China.

**Keywords:** baduanjin, balance, fall, meta-analysis, middle-aged and elderly people, protocol, systematic review

## Abstract

**Background:**

The risk of fall seriously affects the health and quality of life of the middle-aged and elderly people, especially the injury and disability caused by fall of the middle-aged and elderly people, which imposes a huge burden on family and social medical care. Baduanjin exercise may be an effective intervention to enhance the muscle strength and stability of lower limbs, improve the balance ability and gait of middle-aged and elderly people, reduce the incidence of falls, improve the quality of life, and promote the health of middle-aged and elderly people. The aim of this study is to summarize evidence and systematically review the efficacy and safety of Baduanjin on the fall and balance function in middle-aged and elderly people.

**Methods:**

We conducted a systematic search of English and Chinese RCTs in the following 8 electronic databases: PubMed, EMBASE, Web of Science, The Cochrane Library, Chinese Biomedical Literature Database (CBM), Chinese National Knowledge Infrastructure (CNKI), Chinese Science and Technology Periodical Database (VIP), Wanfang Database, from their respective dates of inception to July 2021. Other resources will be searched if necessary. The primary outcome is the fall rate in middle-aged and elderly people and the secondary outcomes include the Single-Leg Standing (SLS) Test, Berg Balance Scale (BBS), Timed Up and Go (TUG) Test. The study selection, data extraction, risk of bias, data synthesis and analysis, reporting biases, and the quality of evidence will be independently conducted by 2 reviewers who use the EndNote X9 software, Cochrane handbook assessment tool, RevMan 5.3 software, a funnel plot and GRADE system.

**Results:**

This study will evaluate the effect of Baduanjin on falls and balance function of middle-aged and elderly people from multiple outcome evaluation indicators such as fall rate, and provide high-quality evidence.

**Conclusion:**

This study will provide evidence for whether Baduanjin has an effect on falls and balance function in middle-aged and elderly people.

**Ethics and dissemination:**

Ethics approval is not required for systematic review, since it does not infringe on personal interests. The results will be submitted to peer-review journals or disseminated at scientific conferences.

## 1 Introduction

A fall is a sudden, involuntary, loss of balance, and fall to the ground or a lower plane. After a fall, about a quarter of middle-aged and elderly people will be restricted by activities or seek medical care.^[1]^ Fall injuries have gradually become a common disease in middle-aged and elderly people. According to the latest statistics of the World Health Organization (WHO), there are an average of 37.3 million falls every year, 10% of the elderly fall at least twice a year, about 646,000 people die from falls, and the direct medical costs are as high as 5 billion RMB.^[2]^ In the United States, about one-third of the elderly fall every year,^[3]^ while the incidence of falls among the elderly in Chinese communities is 14.3%.^[4]^

As they age, the elderly is affected by osteoporosis, chronic degenerative diseases, sleep disorders, motor performance deterioration, cognitive impairment and other physical qualities that change their function, decrease their balance ability, and make them more likely to fall when walking or standing. A systematic review showed that falls in the elderly were also significantly associated with living alone, with social isolation and loneliness as risk factors.^[5]^ But falls are not limited to the elderly, their occurrence is gradually getting younger and more common in women.^[6]^ The occurrence of falls is not only an accident, but is the result of the interaction of internal modifiable factors such as balance and gait, as well as external environmental factors such as tripping and slipping, among which the decline of balance ability is one of the main risk factors. Studies have suggested that balance function training therapy can effectively reduce the risk of falls in middle-aged and elderly people.^[7]^

Falls can not only cause soft tissue injury, fracture, joint dislocation, concussion and other physical injuries, but also may cause depression, irritability, anxiety, loss of self-confidence, and other negative psychology in middle-aged and elderly people, affecting the quality of life, and seriously threatening the life and health of middle-aged and elderly people, which is the second leading cause of accidental injuries and death.^[2]^ Middle-aged and elderly people with a history of falling will limit their activity level because of the fear of falling, which will lead to the decline of physical function and activity ability, and this decline in physical function will increase the risk of falling, and the 2 have adverse effects on each other. With the rapid growth of the population and the aggravation of the aging problem, the huge economic burden of injuries caused by falls on society is foreseeable. At present, there is no effective solution to the status quo of falls in middle-aged and elderly people, and based on the characteristics of high incidence of fall, large economic burden and preventable intervention in the middle-aged and elderly people, it is particularly important to actively seek prevention and treatment measures for falls.

The expert consensus points out that the main method to prevent falls in middle-aged and elderly people includes physical exercise,^[8]^ and there is evidence that well-designed exercise programs can reduce fall rate of middle-aged and elderly people living in the community.^[9]^ Taijiquan, Baduanjin, and Yijinjing are all traditional exercise methods, also known as the “Dao Yin.” Through active exercise and breathing, the body and mind are synchronized, stimulate the potential function of the human body, improve muscle strength and the stability of the body, and enhance the body's adaptability and repair ability to the external environment, so as to achieve the purpose of prevention and treatment of disease. At present, there are many studies on the effect of traditional exercise methods on falls of middle-aged and elderly people, but Taijiquan is relatively complex, difficult to remember, difficult to master, and long practice time-consuming. In recent years, the study on Baduanjin has gradually increased, and it has been found that Baduanjin can significantly improve body's physical function, decrease anxiety and depression, sleep disorders, and other aspects.^[10–12]^ Baduanjin is a traditional fitness exercise composed of 8 movements, easy to learn. It is a low-intensity aerobic exercise that combines body posture, breathing, and mental activities, through slow body movement to stretch the whole body musculoskeletal. The movement of Baduanjin is simple and gentle, with the characteristics of soft and slow, tight and coherent, relaxed, and dynamic and static combination. It is suitable for all ages to practice, especially the middle-aged and elderly people.^[13]^ A systematic review^[14]^ has confirmed that Baduanjin is effective for fall and balance function of middle-aged and elderly people, but the quality of the research is low, and there is a lack of consistency in the outcome evaluation index of fall, leading to some controversy in the conclusion.

Therefore, it is necessary to conduct a high-quality systematic review study on the effects of Baduanjin on fall and balance function in middle-aged and elderly people. The protocol of this systematic review is proposed to point out the direction for follow-up research, in order to provide higher methodological quality evidence to confirm the efficacy of Baduanjin. The protocol of this systematic review has been registered on PROSPERO (http://www.crd.york.ac.uk/PROSPERO), registration number: CRD42021231974.

## 2 Methods

The protocol was designed in strict accordance with the guideline of Preferred Reporting Items for Systematic Review and Meta-Analysis Protocols (PRISMA- P) 2015,^[15]^ and the review will follow this protocol.

### 2.1 Inclusion criteria

#### 2.1.1 Type of studies

All the randomized controlled trials (RCTs) with or without allocation concealment or blindness of Baduanjin on the fall and balance function in middle-aged and elderly people.

#### 2.1.2 Participants

Subjects included in the study should meet the following criteria: Middle-aged and elderly people with age > 45 years as defined by the WHO; The study clearly reported that subjects have a certain risk of falling, whether in a healthy or frail state; The gender, race ,or nationality of the subjects is not restricted; and The living environment of the study subjects is unlimited.

#### 2.1.3 Interventions

The experimental group uses Baduanjin to improve balance function or reduce falls. The specific implementation plan of Baduanjin, such as intervention duration, practice frequency, or genre, is not limited; the interventions received by the control group can include low-intensity exercise, cognitive education training, and other forms of exercise, or not accept any intervention. If there are other interventions, it is required to be consistent between the experimental group and control group.

### 2.2 Outcome measures

#### 2.2.1 Primary outcome

In the meta-analysis, the primary outcome measure to evaluate the effect of Baduanjin on fall and balance function in middle-aged and elderly people is fall rate.^[16]^

#### 2.2.2 Secondary outcomes

##### 2.2.2.1 Single-leg standing (SLS) test

This test is used to evaluate static balance.^[17]^ The subjects were asked to stand on one leg and the other leg bent to 90 degrees. If the raised foot touches the standing leg or the ground, or the subject's standing leg keeps jumping or even requires hand support, the test is terminated and time is recorded. The subjects were tested with 1 eye opening test and 1 eye closing test, repeated 3 times and recorded the best time. Time values<30 seconds when open eyes, the balance was impaired.^[18]^ According to a large normative study, people under the age of 70 should be able to maintain this position for 30 seconds, while those over 70 for an average of 21 seconds and those over 80 for an average of 9 seconds.^[19]^

##### 2.2.2.2 Berg balance scale (BBS)

BBS is the most commonly used scale to evaluate the dynamic and static balance of adults, and it is also the gold standard.^[20,21]^ In almost all balance tests that can identify the risk of falling, BBS has a specificity of up to 77%.^[22]^ The scale divides the balance function into 14 items from easy to difficult for examination, and observes the performance of subjects in various functional activities under sitting and standing positions. Each item was scored on a scale of 0 to 4, with a total score ranging from 0 to 56, with higher scores indicating better balance. The cutoff score is 46, with a score less than 46 indicating a risk of falling.^[23]^ Because BBS evaluates many factors that affect patients’ physical balance, the evaluation is more complex and time-consuming than other scales.

##### 2.2.2.3 Timed up and go (TUG) test

This test is a rapid and quantitative method to evaluate functional walking ability for dynamic balance and mobility.^[24,25]^ Subjects were asked to sit on the arm-backed chairs, then stand up, walk 3 m forward to a marked area, and then return and sit on the chair again. The total time taken to complete this series of actions is the test result. The test is repeated once and the result is recorded. The cutoff value for subjects with a high risk of falling is 13.5 seconds, with > 13.5 seconds being considered to indicate a high risk of falling.^[26]^

### 2.3 Exclusion criteria

The following will be excluded: Nonrandomized controlled trial studies; The full text of the literature is not available; Duplicate publications; The research data are missing or cannot be extracted; and The research that is not rigorously designed or statistically inappropriate.

### 2.4 Search strategy

#### 2.4.1 Electronic searches

The following 8 electronic databases: PubMed, EMBASE, Web of Science, The Cochrane Library, Chinese Biomedical Literature Database (CBM), Chinese National Knowledge Infrastructure (CNKI), Chinese Science and Technology Periodical Database (VIP), Wanfang Database will be searched from their respective dates of inception to July 2021. Other resources will be searched from RCT registration websites (http://www.Clinicaltrials.gov and http://www.chictr.org.cn/), Google scholar (http://scholar.google.com), and Baidu scholar (http://xueshu.baidu.com/).

When PubMed database is used for search, it is carried out in the form of MeSH terms combined with free terms. Other databases are searched with reference to PubMed. The detailed search strategies are summarized in Table 1.

**Table 1:** Search strategy in PubMed.

**#1**	Baduanjin[Tiab] OR Baduanjin exercise[Tiab] OR eight-section Brocade[Tiab] OR traditional health Qigong[Tiab] OR setting-up exercise[Tiab] OR Qigong[Tiab] OR eight section Brocade[Tiab] OR regimen[Tiab] OR rehabilitation exercise[Tiab]
**#2**	Postural Balance[MeSH]) OR balance[Tiab] OR Posture Equilibrium[Tiab] OR Balance, Postural[Tiab] OR Musculoskeletal Equilibrium[Tiab] OR Accidental Falls[Tiab] OR Falls[Tiab] OR Falling[Tiab] OR Slip[Tiab] OR Fall[Tiab]
**#3**	elders[Tiab] OR elderly[Tiab] OR older[Tiab] OR elder[Tiab] OR middle-aged[MeSH] OR middle-age[Tiab]
**#4**	Randomized Controlled Trials as Topic[MeSH] OR Clinical Trials, Randomized[Tiab] OR Trials, Randomized Clinical[Tiab] OR Controlled Clinical Trials, Randomized[Tiab] OR Randomized controlled trial[Tiab] OR controlled clinical trial[Tiab] OR randomized[Tiab] OR placebo[Tiab] OR randomly[Tiab] OR trial[Tiab] OR groups[Tiab]
**#5**	#1 AND #2 AND #3 AND #4

### 2.5 Data collection and analysis

#### 2.5.1 Study selection

**Figure 1:**
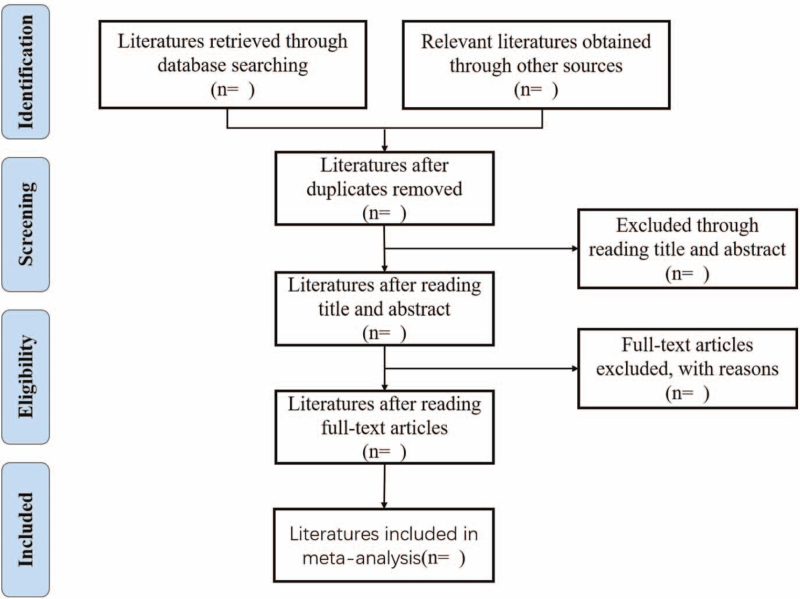
Flow diagram of the study screening.

The researcher imported all the retrieved literatures into the software EndNote X9, and deleted all duplicate literatures. Two reviewers independently screened the literature according to the inclusion criteria and exclusion criteria. First, they read the title of the literature. After excluding the obviously irrelevant literature, they further read the abstract and full text to determine whether the literature was included or not, and cross checked it. Differences, if any, shall be resolved through discussion or negotiation by a third party. Information that is not identified but important to the research can be obtained by contacting the original research author via email or telephone. The research screening process is shown in Figure 1.

### 2.6 Data extraction

Data extraction was conducted independently by 2 reviewers in accordance with the preset standardized data extraction form. The extracted information includes the following contents: the basic information included in the study, such as the research title, first author, published journal, etc; baseline characteristics of the study subjects and intervention measures; the key elements of bias risk assessment; outcome indicators and outcome measurement data concerned. Then, the 2 reviewers cross check the extracted information, and in case of any disagreement, it is resolved through discussion or negotiation by a third party.

### 2.7 Assessment of risk of bias in included studies

The risk of bias in the included studies was independently evaluated by 2 reviewers, and the results were cross-checked. The risk of bias is evaluated using the RCT bias risk assessment tool recommended by Cochrane handbook 5.1.0 (https://training.cochrane.org/handbook).^[27]^ The contents include random sequence generation, allocation concealment, blinding, incomplete outcome data, selective outcome reporting, and other biases. The quality of evidence is assessed using “low risk of bias,” “high risk of bias,” or “unclear” (not enough information to determine whether there is high risk or low risk). If the literature fully meets the above criteria of “low risk of bias,” it indicates that the possibility of various biases is the least and the quality is the highest. Partially satisfied, indicating a moderate possibility of bias and a medium quality; Completely inconsistent, indicating a high risk of bias and low quality.

### 2.8 Data synthesis and analysis

RevMan5.3 software was used for statistical analysis. Mean difference (MD) was used as the statistic of effect analysis for continuous data, and risk ratio (RR) was used as the statistic of effect analysis for dichotomous variables. 95% confidence interval (95% CI) was provided for each effect size. The heterogeneity among the included study results was analyzed by χ^2^ test (α = 0.1), and the magnitude of heterogeneity was quantitatively determined by combining with *I*^2^. If *P* ≥ .1 and I^2^ ≤50%, it means that there was no statistical heterogeneity among the study results, then a fixed-effect model was used for meta-analysis. If *P* < .1, *I*^2^ > 50%, indicating statistical heterogeneity among the study results, the source of heterogeneity was further analyzed. After excluding the influence of obvious clinical heterogeneity, a random-effect model was used for meta-analysis. The level of meta-analysis was set as α = 0.05. Significant clinical heterogeneity was treated by descriptive analysis.

### 2.9 Additional analysis

Subgroup analysis and sensitivity analysis will be planned to do if necessary. Subgroup analysis can be performed according to the type of subjects such as age, gender, and the intervention time of Baduanjin to solve the potential heterogeneity and inconsistency. Sensitivity analysis is mainly to verify the robustness and reliability of the study results in the meta-analysis, and to evaluate the methodological quality, study quality, sample size, impact of missing data, and the impact of analysis methods on the review results. It is also an indirect method to analyze heterogeneity.

### 2.10 Assessment of reporting biases

A funnel plot will be used to assess reporting bias for each study in case that more than 10 eligible studies were included. The Begg test and Egger test will be used to evaluate the symmetry of the funnel plot. If it is not, discuss the source of the bias or try to explain the asymmetry of the funnel plot.^[28]^

### 2.11 Quality of evidence

Two reviewers used the Grades of Recommendations Assessment, Development and Evaluation (GRADE) system^[29]^ (http://www.gradeworkinggroup.org) to independently assess the quality of evidence in the included studies. The limitations in study, inconsistency of results, indirectness of evidence, imprecision and reporting bias will be assessed. The GRADE system divides the quality of evidence into 4 grades: “high, medium, low and very low,” and recommendation into 2 grades: “strong recommendation” and “weak recommendation.”

### 2.12 Ethics and dissemination

The review does not infringe on personal interests and therefore does not require ethical approval. The final study results will be published in peer-reviewed journals or disseminated through conferences.

### 2.13 Patient and public involvement

No patient will be involved in the systematic review. However, the results will be disseminated to people with poor balance and high risk of falls. We only use data that existed in studies published.

## 3 Discussion

Fall has become a common threat to the health of middle-aged and elderly people. Studies have found that the body's balance ability will decline with age, leading to an increased risk of falls.^[30,31]^ At present, there are no clear prevention and treatment measures for falls caused by the decline of balance function in clinical practice, and the key role of exercise in falls is mainly emphasized.^[32]^

Baduanjin, as an excellent traditional body-building method in China, can adjust the body's various functions and the joint mobility as a whole, delay the function decline of the middle-aged and elderly people, and promote their health.^[33]^ Baduanjin is also popular among the majority of middle-aged and elderly people because of its characteristics of slow and soft, coordinated and coherent, and light movement. At present, only some studies with low level of evidence have confirmed the positive effect of Baduanjin exercise on balance function and fall prevention in middle-aged and elderly people.^[34–36]^

The effect of Baduanjin on falls in middle-aged and elderly people is unclear. Hence, the protocol aimed to design a systematic review study to verify the effect of Baduanjin and provide higher-quality evidence, and analyze the specific effect of Baduanjin on balance function of middle-aged and elderly people. If the study can confirm its curative effect, it will help to reduce the fall rate of middle-aged and elderly people, provide a reliable basis for Baduanjin exercise, and popularize its application in the general population.

## Author contributions

Yao Xiao and Qin Luo contributed equally to the work as first authors. All authors approved the final version of this protocol.

**Conceptualization:** Yang Yun Yu, Miao Wu.

**Data curation:** Qin Luo.

**Formal analysis:** Yao Xiao.

**Funding acquisition:** Yan Zhao.

**Methodology:** Miao Wu.

**Project administration:** Yao Xiao, Qin Luo.

**Resources:** Jun Yu Luo.

**Software:** Qin Luo.

**Supervision:** Biwei Cao.

**Validation:** Wei Bi Cao, Jun Yu Luo.

**Visualization:** Yang Yun Yu.

**Writing – original draft:** Yao Xiao, Qin Luo.

**Writing – review & editing:** Jing Zhou.
